# Risk factors for mortality in patients following total hip arthroplasty and hemiarthroplasty due to femoral neck fractures^☆^

**DOI:** 10.1016/j.jor.2025.12.067

**Published:** 2025-12-31

**Authors:** Itay Ron, Lizi Tamam, Bezalel Peskin, Nabil Ghrayeb, Doron Norman, Jacob Shapira

**Affiliations:** a The Ruth and Bruce Rappaport Faculty of Medicine, Technion - Israel Institute of Technology, Haifa, Israel; b Orthopedic Department, Rambam Medical Center, Haifa, Israel

## Abstract

**Background:**

Femoral neck fractures (FNF) in older adults are frequently managed with either total hip arthroplasty (THA) or hemiarthroplasty (HA). Despite improvements in surgical techniques, mortality rates after hip fracture surgery remain high. Identifying predictors of early mortality may enhance surgical decision-making, optimize perioperative management, and improve patient outcomes.

**Purpose:**

The purpose of this study was to determine the short- and mid-term mortality rates after THA and HA for FNF, to identify clinical, demographic, and laboratory factors associated with 30-, 90-, and 180-day mortality, and to establish clinically relevant cutoff thresholds for significant continuous variables to stratify risk.

**Patients and methods:**

We retrospectively reviewed 2379 consecutive patients treated for sub-capital FNF at a tertiary trauma center between [insert study years]. Of these, 831 underwent THA and 1548 underwent HA. Mortality was assessed at 30, 90, and 180 days postoperatively. Demographic, clinical, and laboratory parameters were analyzed using univariate and multivariate logistic regression models. Receiver operating characteristic (ROC) curve analysis was performed to identify optimal cutoff thresholds for significant continuous predictors.

**Results:**

Among THA patients, mortality was 1.4 % at 30 days, 3.4 % at 90 days, and 5.1 % at 180 days. Postoperative albumin ≤2.85 g/dL predicted 30-day mortality, while C-reactive protein (CRP) > 19.15 mg/dL was independently associated with mortality at 90 and 180 days. Among HA patients, mortality was 6.6 % at 30 days, 12.9 % at 90 days, and 17.6 % at 180 days. Predictors of 30-day mortality included white blood cell count (WBC) > 14.48 × 10^9^/L, albumin <3.55 g/dL, and Charlson Comorbidity Index (CCI) > 7.5. At 90 and 180 days, age >83.65 and > 89.34 years, WBC >13.49 × 10^9^/L, albumin <3.35–3.45 g/dL, creatinine >1.08 mg/dL, and CCI >6.5 were associated with higher mortality risk.

**Conclusions:**

This study identified several laboratory and clinical markers that predict short- and mid-term mortality following hip arthroplasty for FNF. Hypoalbuminemia, elevated inflammatory markers, renal dysfunction, and high comorbidity burden were consistent risk factors. Incorporating these parameters into preoperative assessment may improve patient selection, perioperative optimization, and shared decision-making.

**Level of Evidence:**

III

## 1 Introduction

Hip fractures are a significant public health issue, due to their high frequency and considerable severity.^1^, ^2^, ^3^ Patients with hip fractures are at a substantial risk for death, health complications, and reduced quality of life.^4^^,^^5^ Displaced femoral neck fractures (FNF) in adults are generally treated with either total or partial hip arthroplasty, depending on the general health and mental status as well as the ambulatory requirements of the patients. Total hip arthroplasty (THA) following femoral neck fracture (FNF) is associated with improved function, increased cost-effectiveness, satisfaction, and a lower reoperation rate compared to hemiarthroplasty (HA).^6^, ^7^, ^8^, ^9^, ^10^ In contrast, THA is also associated with longer operation times, higher blood loss, and a higher dislocation rate than hemiarthroplasty.^11^^,^^12^

Previous studies investigating surgical management following FNF have found an association between increased mortality risk and older age, higher American Society of Anesthesiologists (ASA) class, lower body mass index (BMI), longer time to surgery, and lower serum hemoglobin levels.^13^, ^14^, ^15^ One-year mortality following surgical management for FNF has been estimated to be approximately 20 %, with no significant change in the past three decades.^16^^,^^17^ Nevertheless, the early mortality rate in patients with FNF is lower following THA than hemiarthroplasty, possibly due to the selection of more morbid patients for the latter.^18^^,^^19^

Still, there is no consensus on the optimal management of displaced FNF in specific patient populations, especially among older active adults. Therefore, the association of THA with improved postoperative outcomes, alongside its longer surgery times and higher complication risks, highlights the need for a tailored treatment algorithm to enhance patient selection. This will ensure optimal outcomes for patients with FNF while minimizing potential risks, including mortality.

Given the high prevalence of sub-capital fractures, their substantial public health impact, and the importance of minimizing repeat surgeries and morbidity, this study aimed to investigate the risk factors for mortality in patients with femoral neck fractures (FNF) treated with either total or partial hip arthroplasty. This study hypothesized that factors related to laboratory measures, overall comorbidity status, and type of operative procedure might be predictive of mortality after subcapital fractures.

## 2 Methods

### 2.1 Patient selection and Defining cohorts

This controlled retrospective study included data records from the orthopedic department in a tertiary trauma center.

Eligible patients were those who suffered from sub-capital fracture and were treated with either total hip arthroplasty or partial hip arthroplasty. Patients were classified based on the type of procedure (THA or HA) and mortality status. Mortality outcomes were evaluated at three distinct postoperative time points: 30 days, 90 days, and 180 days after surgery. Patients were excluded from the study if their primary injury was a femoral neck fracture, which is not a subcapital fracture. Furthermore, patients were excluded if their initial treatment was other than total or hemi-arthroplasty.

The hospital database was used to identify the relevant patients, and the search was conducted through the MDClone ADAMS system, which is integrated with our electronic medical records. This system provides a self-service data analytics environment that enables the generation of complex search queries and allows easy access to all retrospective data within the hospital.

The evaluated demographic variables were age, gender, BMI, smoking status, presence of vascular disease, dialysis status, nutritional status (i.e., albumin, lymphocytes, leukocytes), pre surgery hemoglobin, culture biopsies, use of antibiotics, estimated glomerular filtration rate (eGFR), blood Creatinine levels, Charlson Comorbidity Index (CCI), and other comorbidities (i.e., s/p CVA, hypertension, myocardial infraction).

Laboratory measures were measured up to 2 weeks before the initial sub-capital fracture.

### 2.2 Statistical analysis

Continuous variables were reported using mean ± standard deviation (SD) for normally distributed variables, and as median with interquartile range (IQR) for non-normally distributed variables. Categorical variables were recorded as counts and percentages. Normality of the quantitative parameters was tested using the Kolmogorov-Smirnov test. As a result of this test, either a parametric (*t*-test) or nonparametric (Mann-Whitney *U* test) test was used to detect differences between the two groups. Differences between the two groups for categorical variables were calculated using the chi-squared or Fisher's exact test. A multivariate logistic regression model was employed to predict risk factors associated with mortality. A receiver operating characteristic (ROC) curve with the area under the curve and 95 % confidence interval was constructed to describe the relationship between sensitivity and the false-positive rate for laboratory measures in identifying patients at risk for mortality after total hip arthroplasty or hemiarthroplasty. For each time point (30, 90, and 180 days), computer-assisted multivariable logistic regression models were created for patients undergoing total hip arthroplasty (THA) and hemiarthroplasty. The models included only variables that were significant in the univariate analysis. Additionally, a Youden index analysis was performed on the continuous variables to determine the optimal threshold. SPSS version 27 was used for all statistical analyses. This research was approved by the Institutional Review Board (IRB) committee. No funding source was involved in this study.

## 3 Results

### 3.1 Demographics

A total of 2379 patients with sub-capital fractures in their medical history and who had been treated with either total hip or partial hip arthroplasty were identified. Among them, 1548 (65 %) patients underwent partial hip arthroplasty, and 831 (35 %) underwent total hip arthroplasty. The overall population included 850 (35.7 %) male patients and 1529 (64.3 %) female patients. 305 patients underwent total hip arthroplasty, while 545 were treated with a partial hip arthroplasty. 526 female patients underwent total hip arthroplasty, and 1003 underwent partial hip arthroplasty. The mean age for patients in the total hip arthroplasty group was 71.8 years, while the mean age in the partial hip arthroplasty group was 82.5 years.

### 3.2 Total hip arthroplasty

Of the 885 patients who underwent total hip arthroplasty, 12 (1.4 %) died within 30 days, 30 (3.4 %) within 90 days, and 45 (5.1 %) within 180 days. Age was significantly higher in the 30-day mortality group (77.5 ± 9.1 vs. 71.7 ± 9.8 years, p = 0.04) but was not associated with mortality at later time points. Several variables were consistently associated with increased mortality across all time points, including chronic obstructive pulmonary disease (30-day: 25.0 % vs. 4.0 %, p = 0.01; 90-day: 16.7 % vs. 3.9 %, p < 0.001; 180-day: 11.1 % vs. 3.9 %, p = 0.021), chronic kidney disease (30-day: 33.3 % vs. 7.7 %, p = 0.01; 90-day: 30.0 % vs. 7.3 %, p < 0.001; 180-day: 31.1 % vs. 6.8 %, p < 0.001), and smoking history (30-day: 33.3 % vs. 11.6 %, p = 0.04; 90-day: 26.7 % vs. 11.3 %, p = 0.011; 180-day: 24.4 % vs. 11.2 %, p = 0.007), **as seen in** Table 1, Table 3, Table 5. Postoperative inflammatory markers were significantly elevated among patients who died, including CRP (30-day: 28.9 ± 12.8 vs. 15.5 ± 8.7 mg/dL, p = 0.003; 90-day: 23.5 ± 10.4 vs. 15.2 ± 8.6, p < 0.001; 180-day: 20.4 [12.7–27.4] vs. 14.9 [7.4–20.5], p = 0.003) and white blood cell count (90-day: 14.9 ± 9.3 vs. 11.8 ± 5.5, p = 0.006; 180-day: 14.2 ± 8.4 vs. 11.9 ± 5.5, p = 0.009).

**Table 1:** Arthroplasty – mortality at 30 Days.

Variable	Alive (n = 873)	Death (n = 12)	P-value
Age (years)	71.7 ± 9.8	77.5 ± 9.1	**0.042**
Length of stay (days)	7.3 ± 6.0	4.4 ± 3.1	0.64
Procedure side -Left	411 (47 %)	3 (25 %)	0.15
Procedure side - Right	462 (53 %)	9 (75 %)	
Hypertension	405 (46.4 %)	7 (58.3 %)	0.41
CVA	30 (3.4 %)	2 (16.7 %)	0.067
COPD	35 (4.0 %)	3 (25.0 %)	**0.012**
Myocardial infarction	49 (5.6 %)	0 (0.0 %)	1.00
Diabetes mellitus	215 (24.6 %)	5 (41.7 %)	0.18
BMI	25.9 ± 4.6 (n = 240)	25.3 ± 6.9 (n = 5)	0.76
HbA1c (%)	7.3 ± 2.3 (n = 76)	10.4 ± 5.6 (n = 2)	0.07
Antibiotics after surgery	N = 762	N = 10	1.00
IV	744 (97.6 %)	10 (100 %)	
PO	18 (2.4 (	0	
Antibiotics before surgery	N = 67	N = 1	NA
IM	19 (28.4 %)	0	
IV	44 (65.7 %)	1	
PO	4 (6.0 %)	0	
Hemoglobin before surgery (g/dL)	12.5 ± 1.7	12.1 ± 1.9	0.42
Hemoglobin after surgery (g/dL)	9.7 ± 1.7	9.4 ± 1.7	0.46
Glucose max 14 days before surgery (mg/dL)	137.6 ± 56.9	163.3 ± 76.5	0.12
Glucose 1 day before surgery (mg/dL)	139.2 ± 60.1	169.9 ± 76.6	0.094
Glucose 1 day after surgery (mg/dL)	146.2 ± 48.9	164.4 ± 94.1	0.41
CRP after surgery (mg/dL)	15.5 ± 8.7 (n = 274)	28.9 ± 12.8 (n = 4)	**0.003**
WBC before surgery (K/μL)	11.5 ± 4.6	11.7 ± 4.9	0.88
WBC after surgery (K/μL)	11.9 ± 5.7	13.0 ± 7.1	0.53
Lymphocytes before surgery (10^1^/μL)	2.6 [1.2–12.5]	3.8 [1.0–16.9]	0.95
Lymphocytes after surgery (10^1^/μL)	2.67 [1.17–13.2]	1.75 [0.71–14.1]	0.43
Albumin before surgery (g/dL)	3.5 ± 0.6	3.5 ± 0.65	0.90
Albumin after surgery (g/dL)	3.12 ± 0.55 (n = 624)	2.44 ± 0.64 (n = 5)	**0.006**
Creatinine (mg/dL)	1.09 ± 0.87	1.47 ± 0.62	0.13
Chronic kidney disease	67 (7.7 %)	4 (33.3 %)	**0.012**
Charlson Comorbidity Index	4.87 ± 1.98	5.09 ± 1.58	0.71
Smoking	101 (11.6 %)	4 (33.3 %)	**0.044**
Revision surgery	91 (10.4 %)	2 (16.7 %)	0.36

**Table 2:** Hemiarthroplasty – mortality at 30 Days.

Variable	Alive (n = 1529)	Death (n = 108)	P-value
Age (years)	82.5 ± 8.2	84.3 ± 8.3	**0.027**
Length of stay (days)	8.3 ± 5.8	8.7 ± 6.7	0.51
Procedure side -Left	771 (50.4 %)	50 (46.3 %)	0.41
Procedure side-Right	758 (49.6 %)	58 (53.7 %)	
Hypertension	903 (59.1 %)	72 (66.7 %)	0.12
CVA	96 (6.3 %)	7 (6.5 %)	0.93
COPD	70 (4.6 %)	6 (5.6 %)	0.64
Myocardial infarction	119 (7.8 %)	14 (13.0 %)	0.057
Diabetes mellitus	391 (25.6 %)	32 (29.6 %)	0.35
BMI	25.7 ± 4.7 (n = 288)	25.6 ± 3.8 (n = 26)	0.98
HbA1c (%)	7.1 ± 2.0 (n = 123)	7.0 ± 2.1 (n = 14)	0.89
Antibiotics after surgery	N = 1059	N = 87	0.93
IV	1016 (95.9 %)	84 (96.6 %)	
PO	42 (4.0 %)	3 (3.7 %)	
SC	1 (0.1 %)	0	
Antibiotics before surgery	N = 128	N = 18	0.77
IM	39 (30.5 %)	4 (22.2 %)	
IV	83 (64.8 %)	13 (72.2 %)	
PO	6 (4.7 %)	1 (5.6 %)	
Hemoglobin before surgery (g/dL)	12.2 ± 1.7	11.7 ± 2.02	**0.02**
Hemoglobin after surgery (g/dL)	9.5 ± 1.6	8.9 ± 1.4	**<0.001**
Glucose max 14 days before surgery (mg/dL)	141.1 ± 48.7	154.3 ± 67.7	**0.01**
Glucose 1 day before surgery (mg/dL)	142.3 ± 50.6	154.1 ± 68.1	**0.03**
Glucose 1 day after surgery (mg/dL)	147.6 ± 59.9	152.9 ± 61.3	0.49
CRP after surgery (mg/dL)	19.06 ± 9.2 (n = 459)	24.2 ± 10.2 (n = 47)	**<0.001**
WBC before surgery (K/μL)	11.7 ± 4.0	12.3 ± 5.8	0.18
WBC after surgery (K/μL)	12.3 ± 4.7	17.2 ± 7.9	**<0.001**
Lymphocytes before surgery (10^1^/μL)	7.3 [1.4–13.8]	6.5 [1.2–12.8]	0.16
Lymphocytes after surgery (10^1^/μL)	7.55 [1.3–13.4]	6.4 [0.94–10.75]	**0.006**
Albumin before surgery (g/dL)	3.43 ± 0.58	3.19 ± 0.63	**<0.001**
Albumin after surgery (g/dL)	2.93 ± 0.52	2.63 ± 0.59	**<0.001**
Creatinine (mg/dL)	1.23 ± 0.94	1.89 ± 1.83	**<0.001**
Chronic kidney disease	177 (11.6 %)	28 (25.9 %)	**<0.001**
Charlson Comorbidity Index	6.04 ± 1.9	6.9 ± 2.25	**<0.001**
Smoking	121 (8.5 %)	35 (16.6 %)	**<0.001**
Revision surgery	43 (2.8 %)	0 (0.0 %)	0.11

**Table 3:** Arthroplasty – mortality at 90 Days.

Variable	Alive (n = 885)	Death (n = 30)	P-value
Age (years)	71.7 ± 9.9	73.9 ± 9.7	0.23
Length of hospital stay (days)	7.2 ± 5.9	8.8 ± 8.3	0.13
procedure side – Left	405 (47 %)	9 (30 %)	0.061
procedure side – Right	450 (53 %)	21 (70 %)	
Hypertension	394 (46.1 %)	18 (60.0 %)	0.13
CVA	26 (3.0 %)	6 (20.0 %)	**<0.001**
COPD	33 (3.9 %)	5 (16.7 %)	**<0.001**
Myocardial infarction	48 (5.6 %)	1 (3.3 %)	1.00
Diabetes mellitus	209 (24.4 %)	11 (36.7 %)	0.13
BMI	25.8 ± 4.4	27.1 ± 7.7	0.31
HbA1c (%)	7.37 ± 2.2 (n = 71)	7.57 ± 3.6 (n = 7)	0.84
Antibiotics after surgery	N = 747	N = 25	0.11
IV	731 (97.9 %)	23 (92 %)	
PO	16 (2.1 %)	2 (8 %)	
Antibiotics before surgery	N = 64	N = 4	0.17
IM	19 (29.7 %)	0	
IV	41 (64.1 %)	4 (100 %)	
PO	4 (6.3 %)	0	
Hemoglobin before surgery (g/dL)	12.5 ± 1.7	12.2 ± 2.3	0.33
Hemoglobin after surgery (g/dL)	9.75 ± 1.7	9.14 ± 1.6	0.065
Glucose 14 days before surgery (mg/dL)	138.1 ± 57.3	135.6 ± 57.3	0.82
Glucose 1 day before surgery (mg/dL)	139.6 ± 60.6	141.2 ± 57.3	0.89
Glucose 1 day after surgery (mg/dL)	146.7 ± 48.9	137.6 ± 67.2	0.50
CRP after surgery (mg/dL)	15.2 ± 8.6	23.5 ± 10.4	**<0.001**
WBC before surgery (K/μL)	11.5 ± 4.56	11.5 ± 4.58	0.97
WBC after surgery (K/μL)	11.8 ± 5.5	14.9 ± 9.3	**0.006**
Lymphocytes before surgery (10^1^/μL)	2.56 [1.2–12.5]	6.2 [1.32–13.25]	0.80
Lymphocytes after surgery (10^1^/μL)	3.04 [1.18–13.3]	1.41 [0.73–7.30]	**0.023**
Albumin before surgery (g/dL)	3.53 ± 0.58	3.17 ± 0.85	**0.004**
Albumin after surgery (g/dL)	3.13 ± 0.55	2.58 ± 0.49	**<0.001**
Creatinine (mg/dL)	1.07 ± 0.83	1.69 ± 1.56	**<0.001**
Chronic kidney disease	62 (7.3 %)	9 (30.0 %)	**<0.001**
Charlson Comorbidity Index	4.84 ± 1.97	5.38 ± 1.92	0.17
Smoking	97 (11.3 %)	8 (26.7 %)	**0.011**
Revision surgery	91 (10.6 %)	2 (6.7 %)	0.76

Markers of poor nutritional and immune status were also associated with mortality. Albumin levels were lower both before and after surgery (pre-op: 90-day: 3.17 ± 0.85 vs. 3.53 ± 0.58, p = 0.004; 180-day: 3.22 ± 0.75 vs. 3.53 ± 0.58, p = 0.003; post-op: 30-day: 2.44 ± 0.64 vs. 3.12 ± 0.55, p = 0.006; 90-day: 2.58 ± 0.49 vs. 3.13 ± 0.55, p < 0.001; 180-day: 2.71 ± 0.54 vs. 3.13 ± 0.55, p < 0.001), and postoperative lymphocyte counts were reduced (90-day: 1.41 [0.73–7.30] vs. 3.04 [1.18–13.3], p = 0.023; 180-day: 1.43 [0.77–5.80] vs. 3.13 [1.18–13.6], p = 0.002). Creatinine levels were significantly higher in the 90- and 180-day mortality groups (90-day: 1.69 ± 1.56 vs. 1.07 ± 0.83 mg/dL; 180-day: 1.68 ± 1.42 vs. 1.06 ± 0.82, both p < 0.001). Cerebrovascular disease was more common among those who died at 90 and 180 days (90-day: 20.0 % vs. 3.0 %, p < 0.001; 180-day: 13.3 % vs. 3.1 %, p < 0.001), and hemoglobin levels were lower in the 180-day group both before (11.8 ± 2.3 vs. 12.5 ± 1.7 g/dL, p = 0.007) and after surgery (8.95 ± 1.96 vs. 9.76 ± 1.70, p = 0.002). Finally, patients who died within 180 days had more extended hospital stays (9.4 ± 8.7 vs. 7.1 ± 5.8 days, p = 0.015) and higher Charlson comorbidity scores (5.86 ± 2.35 vs. 4.80 ± 1.92, p = 0.002).

### 3.3 Hemiarthroplasty

Among 1637 patients who underwent hemiarthroplasty, mortality occurred in 108 (6.6 %) at 30 days, 211 (12.9 %) at 90 days, and 288 (17.6 %) at 180 days. Across all time points, patients who died were significantly older (30-day: 84.3 ± 8.3 vs. 82.5 ± 8.2, p = 0.027; 90-day: 83.9 ± 8.7 vs. 82.4 ± 8.1, p = 0.016; 180-day: 83.5 ± 8.8 vs. 82.4 ± 8.0, p = 0.050), **as seen in** Table 2, Table 4, Table 6. Hemoglobin levels were significantly lower in the mortality groups, both preoperatively (30-day: 11.7 ± 2.0 vs. 12.2 ± 1.7, p = 0.020; 90-day: 11.73 ± 2.06 vs. 12.19 ± 1.7, p < 0.001; 180-day: 11.65 ± 2.06 vs. 12.24 ± 1.70, p < 0.001) and postoperatively (30-day: 8.9 ± 1.4 vs. 9.5 ± 1.6, p < 0.001; 90-day: 8.95 ± 1.57 vs. 9.56 ± 1.56, p < 0.001; 180-day: 8.98 ± 1.56 vs. 9.59 ± 1.55, p < 0.001). Postoperative inflammatory markers were significantly elevated in patients who died, including CRP (30-day: 24.2 ± 10.2 vs. 19.1 ± 9.2 mg/dL, p < 0.001; 90-day: 22.6 ± 10.3 vs. 18.6 ± 9.1, p < 0.001; 180-day: 20.7 [14.6–28.0] vs. 18.9 [11.9–25.6], p = 0.036) and white blood cell count (30-day: 17.2 ± 7.9 vs. 12.3 ± 4.7, p < 0.001; 90-day: 15.5 ± 7.1 vs. 12.2 ± 4.6, p < 0.001; 180-day: 14.9 ± 7.1 vs. 12.2 ± 4.4, p < 0.001). Postoperative lymphocyte counts were lower in the mortality groups (30-day: 6.4 [0.94–10.75] vs. 7.55 [1.3–13.4], p = 0.006; 90-day: 5.9 [0.97–11.0] vs. 7.7 [1.38–13.6], p < 0.001; 180-day: 5.5 [0.98–11.0] vs. 7.9 [1.41–13.8], p < 0.001), as were albumin levels both preoperatively (30-day: 3.19 ± 0.63 vs. 3.43 ± 0.58, p < 0.001; 90-day: 3.30 ± 0.61 vs. 3.43 ± 0.58, p = 0.009; 180-day: 3.28 ± 0.60 vs. 3.45 ± 0.58, p < 0.001) and postoperatively (30-day: 2.63 ± 0.59 vs. 2.93 ± 0.52, p < 0.001; 90-day: 2.68 ± 0.55 vs. 2.94 ± 0.52, p < 0.001; 180-day: 2.65 ± 0.54 vs. 2.97 ± 0.51, p < 0.001). Creatinine was significantly higher among patients who died (30-day: 1.89 ± 1.83 vs. 1.23 ± 0.94 mg/dL, p < 0.001; 90-day: 1.82 ± 1.63 vs. 1.19 ± 0.88, p < 0.001; 180-day: 1.69 ± 1.51 vs. 1.18 ± 0.87, p < 0.001), and kidney disease was more prevalent (30-day: 25.9 % vs. 11.6 %; 90-day: 24.6 % vs. 10.7 %; 180-day: 21.5 % vs. 10.6 %; all p < 0.001). The Charlson comorbidity index was significantly higher in each mortality group (30-day: 6.9 ± 2.25 vs. 6.04 ± 1.9, p < 0.001; 90-day: 6.98 ± 2.34 vs. 5.95 ± 1.78, p < 0.001; 180-day: 6.88 ± 2.36 vs. 5.91 ± 1.72, p < 0.001). Elevated glucose levels 24 h before surgery were also associated with 30-day mortality (154.1 ± 68.1 vs. 142.3 ± 50.6 mg/dL, p = 0.03). Additional risk factors were identified at later time points, including longer hospitalization duration (90-day: 9.5 ± 7.2 vs. 8.1 ± 5.6 days, p < 0.001; 180-day: 9.87 ± 8.72 vs. 7.96 ± 4.9, p < 0.001), history of myocardial infarction (90-day: 12.3 % vs. 7.5 %, p = 0.017; 180-day: 12.2 % vs. 7.3 %, p = 0.006), diabetes mellitus (180-day: 30.6 % vs. 24.8 %, p = 0.044), COPD (180-day: 7.6 % vs. 4.0 %, p = 0.008), and smoking (90-day: 16.6 % vs. 8.5 %, p < 0.001; 180-day: 14.2 % vs. 8.5 %, p = 0.003).

**Table 4:** Hemiarthroplasty- mortality at 90 Days.

Variable	Alive (n = 1426)	Death (n = 211)	P-value
Age (years)	82.4 ± 8.1	83.9 ± 8.73	**0.016**
Length of stay (days)	8.11 ± 5.63	9.5 ± 7.2	**<0.001**
Procedure side – Left	721 (50.6 %)	100 (47.4 %)	0.39
Procedure side – Right	705 (49.4 %)	111 (52.6 %)	
Hypertension	839 (58.8 %)	136 (64.5 %)	0.12
CVA	90 (6.3 %)	13 (6.2 %)	0.93
COPD	62 (4.3 %)	14 (6.6 %)	0.14
Myocardial infarction	107 (7.5 %)	26 (12.3 %)	**0.017**
Diabetes Mellitus	364 (25.5 %)	59 (28.0 %)	0.45
BMI	25.7 ± 4.58	25.6 ± 4.94	0.87
HbA1c (%)	7.15 ± 1.9 (n = 109)	6.76 ± 2.07 (n = 28)	0.36
Antibiotics after surgery	N = 975	N = 171	0.47
IV	933 (95.7 %)	167 (97.7 %)	
PO	41 (4.2 %)	4 (2.3 %)	
SC	1 (0.1 %)	0	
Antibiotics before surgery	N = 119	N = 27	0.60
IM	37 (31.1 %)	6 (22.2 %)	
IV	76 (63.9 %)	20 (74.1 %)	
PO	6 (5.0 %)	1 (3.7 %)	
Hemoglobin before surgery (g/dL)	12.19 ± 1.7	11.73 ± 2.06	**<0.001**
Hemoglobin after surgery (g/dL)	9.56 ± 1.56	8.95 ± 1.57	**<0.001**
Glucose max 14 days before surgery (mg/dL)	141.2 ± 48.9	147.1 ± 58.9	0.13
Glucose 1 day before surgery (mg/dL)	142.6 ± 50.8	146.3 ± 59.1	0.37
Glucose 1 day after surgery (mg/dL)	148.0 ± 60.6	148.4 ± 56.1	0.94
CRP after surgery (mg/dL)	18.56 ± 9.09	22.55 ± 10.28	**<0.001**
WBC before surgery (K/μL)	11.71 ± 3.94	12.28 ± 5.35	0.073
WBC after surgery (K/μL)	12.22 ± 4.59	15.52 ± 7.10	**<0.001**
Lymphocyte before surgery (10^1^/μL)	7.45 [1.44–13.9]	6.55 [1.30–12.65	0.11
Lymphocytes after surgery (10^1^/μL)	7.70 [1.38–13.6]	5.90 [0.97–11.0]	**<0.001**
Albumin before surgery (g/dL)	3.43 ± 0.58	3.30 ± 0.61	**0.009**
Albumin after surgery (g/dL)	2.94 ± 0.52	2.68 ± 0.55	**<0.001**
Creatinine (mg/dL)	1.19 ± 0.88	1.82 ± 1.63	**<0.001**
Chronic kidney disease	153 (10.7 %)	52 (24.6 %)	**<0.001**
Charlson comorbidity index	5.95 ± 1.78	6.98 ± 2.34	**<0.001**
Smoking	121 (8.5 %)	35 (16.6 %)	**<0.001**
Revision surgery	39 (2.7 %)	4 (1.9 %)	0.65

**Table 5:** Arthroplasty – mortality at 180 Days.

Variable	Alive (n = 840)	Death (n = 45)	P-value
Age (years)	71.6 ± 9.8	74.0 ± 9.9	0.11
Length of stay (days)	7.1 ± 5.8	9.4 ± 8.7	**0.015**
Procedure side-Left	395 (47 %)	19 (42 %)	0.53
Procedure side-Right	445 (53 %)	26 (58 %)	
Hypertension	386 (46.0 %)	26 (57.8 %)	0.12
CVA	26 (3.1 %)	6 (13.3 %)	**<0.001**
COPD	33 (3.9 %)	5 (11.1 %)	**0.021**
Myocardial Infarction	47 (5.6 %)	2 (4.4 %)	1.00
Diabetes Mellitus	204 (24.3 %)	16 (35.6 %)	0.088
BMI	25.8 ± 4.5	26.5 ± 6.4	0.53
HbA1c (%)	7.29 ± 2.2 (n = 69)	8.17 ± 3.42 (n = 9)	0.31
Antibiotics after surgery	N = 732	N = 40	0.24
IV	716 (97.8 %)	38 (95 %)	
PO	16 2.2 %)	2 (5 %)	
Antibiotics before surgery	N = 62	N = 6	0.19
IM	19 (30.6 %)	0	
IV	39 (62.9 %)	6 (100 %)	
PO	4 (6.5 %)	0	
Hemoglobin before surgery (g/dL)	12.5 ± 1.7	11.8 ± 2.3	**0.007**
Hemoglobin after surgery (g/dL)	9.76 ± 1.70	8.95 ± 1.96	**0.002**
Glucose 14 days before surgery (mg/dL)	138.8 ± 57.1	140.8 ± 61.3	0.73
Glucose 1 day before surgery (mg/dL)	139.2 ± 60.2	148.1 ± 64.6	0.36
Glucose 1 day after surgery (mg/dL)	146.6 ± 47.8	143.2 ± 74.2	0.75
CRP after surgery (mg/dL)	14.9 [7.37–20.5]	20.4 [12.7–27.4]	**0.003**
WBC before surgery (K/μL)	11.55 ± 4.55	11.1 ± 4.79	0.51
WBC after surgery (K/μL)	11.86 ± 5.5	14.18 ± 8.37	**0.009**
Lymphocytes before surgery (10^1^/μL)	2.6 [1.2–12.5]	4.5 [0.96–13.1]	0.65
Lymphocytes after surgery (10^1^/μL)	3.13 [1.18–13.6]	1.43 [0.77–5.80]	**0.002**
Albumin before surgery (g/dL)	3.53 ± 0.58	3.22 ± 0.75	**0.003**
Albumin after surgery (g/dL)	3.13 ± 0.55	2.71 ± 0.54	**<0.001**
Creatinine (mg/dL)	1.06 ± 0.82	1.68 ± 1.42	**<0.001**
Chronic kidney disease	57 (6.8 %)	14 (31.1 %)	**<0.001**
Charlson score	4.80 ± 1.92	5.86 ± 2.35	**0.002**
Smoking	94 (11.2 %)	11 (24.4 %)	**0.007**
Revision	89 (10.6 %)	4 (8.9 %)	1.00

**Table 6:** Hemiarthroplasty– mortality at 180 Days.

Variable	Alive; n = 1349	Death; n = 288	p-value
Age	82.4 ± 8.02	83.5 ± 8.8	0.05
Length of Stay	7.96 ± 4.9	9.87 ± 8.72	**<0.001**
Procedure side-Left	675 (50.0 %)	146 (51 %)	0.84
Procedure side-Right	674 (50 %)	142 (49 %)	
Hypertension	793 (58.8 %)	182 (63.2 %)	0.17
CVA	87 (6.4 %)	16 (5.6 %)	0.57
COPD	54 (4.0 %)	22 (7.6 %)	**0.008**
Myocardial Infarction	98 (7.3 %)	35 (12.2 %)	**0.006**
Diabetes Mellitus	335 (24.8 %)	88 (30.6 %)	**0.044**
BMI	25.7 ± 4.55	25.3 ± 4.93	0.51
HbA1c (%)	7.16 ± 2.04	6.81 ± 1.91	0.38
Antibiotics after surgery	N = 919	N = 227	P = 0.48
IV	879 (95.6 %)	221 (97.4 %)	
PO	39 (4.2 %)	6 (2.6 %)	
SC	1 (0.1 %)	0	
Antibiotics before surgery	N = 110	N = 36	0.32
IM	36 (32.7 %)	7 (19.4 %)	
IV	69 (62.7 %)	27 (75.0 %)	
PO	5 (4.5 %)	2 (5.6 %)	
Hemoglobin before surgery (g/dL)	12.24 ± 1.70	11.65 ± 2.06	**<0.001**
Hemoglobin after surgery (g/dL)	9.59 ± 1.55	8.98 ± 1.56	**<0.001**
Glucose 14 days before surgery	141.6 ± 49.3	144.1 ± 54.9	0.46
Glucose 1 day before surgery	142.9 ± 51.4	144.1 ± 55.1	0.75
Glucose 1 day after surgery	148.3 ± 61.4	147.1 ± 53.7	0.82
CRP after surgery (mg/dL)	18.9 [11.9–25.6]	20.7 [14.6–28.0]	**0.036**
WBC before surgery (K/μL)	11.7 ± 3.93	12.09 ± 5.03	0.18
WBC after surgery (K/μL)	12.2 ± 4.43	14.9 ± 7.1	**<0.001**
Lymphocytes before surgery (10^1^/μL)	7.4 [1.5–13.9]	6.75 [1.24–12.8]	0.088
Lymphocytes after surgery (10^1^/μL)	7.9 [1.41–13.8]	5.5 [0.98–11.0]	**<0.001**
Albumin before surgery (g/dL)	3.45 ± 0.58	3.28 ± 0.60	**<0.001**
Albumin after surgery (g/dL)	2.97 ± 0.51	2.65 ± 0.54	**<0.001**
Creatinine (mg/dl)	1.18 ± 0.87	1.69 ± 1.51	**<0.001**
Kidney Disease	143 (10.6 %)	62 (21.5 %)	**<0.001**
Charlson Score	5.91 ± 1.72	6.88 ± 2.36	**<0.001**
Smoking Diagnosis	115 (8.5 %)	41 (14.2 %)	**0.003**
Revision	35 (2.6 %)	8 (2.8 %)	0.86

### 3.4 Regression analysis

In the THA group, at 30 days postoperatively, albumin levels ≤2.85 g/dL were associated with a significantly increased mortality risk (OR = 0.112, 95 % CI: 0.012–1.008, p = 0.051). At 90 days, CRP >19.15 mg/dL was a strong predictor of mortality (OR = 4.486, 95 % CI: 1.243–16.193, p = 0.022). At 180 days, CRP >19.15 mg/dL remained independently associated with increased mortality (OR = 4.458, 95 % CI: 1.902–10.449, p < 0.001).

In the hemiarthroplasty group, the 30-day mortality model identified three significant predictors: WBC >14.48 x10^9^/L (OR = 3.523, 95 % CI: 2.047–6.062, p < 0.001; AUC = 0.702), albumin <3.55 g/dL (OR = 0.364, 95 % CI: 0.192–0.693, p = 0.002; AUC = 0.615), and Charlson score >7.5 (OR = 1.768, 95 % CI: 0.987–3.168, p = 0.056; AUC = 0.618). For 90-day mortality, all five dichotomized variables remained significant: age >83.65 years (OR = 1.531, p = 0.041), WBC >13.49, pre-operative albumin <3.35, creatinine >1.08 mg/dL, and Charlson score >6.5. Similarly, at 180 days, mortality was associated with: age >89.34 (OR = 1.949, p = 0.013), WBC >13.49 (OR = 2.075, p < 0.001), pre-operative albumin <3.45 (OR = 0.510, p = 0.002), postoperative albumin <2.85 (OR = 0.432, p < 0.001), and Charlson score >6.5 (OR = 1.990, p = 0.001).

## 4 Discussion

This study evaluated risk factors for mortality in patients with femoral neck fractures treated with either total or partial hip arthroplasty. Multivariable regression analyses were performed separately for 30-day, 90-day, and 180-day mortality time points. In the total hip arthroplasty group, low postoperative albumin levels (≤2.85 g/dL) were associated with increased 30-day mortality, while elevated postoperative CRP levels (>19.15 mg/dL) were associated with mortality at both 90 and 180 days. In the hemiarthroplasty group, 30-day mortality was predicted by elevated white blood cell (WBC) counts (>14.48 × 10^9/L), low albumin (<3.55 g/dL), and a Charlson comorbidity score greater than 7.5. For both 90- and 180-day mortality, similar risk factors emerged, including older age (>83.65 and > 89.34 years, respectively), elevated WBC (>13.49 × 10^9^/L), low albumin (<3.35–3.45 g/dL), impaired renal function (creatinine >1.08 mg/dL), and Charlson score >6.5.

This study examined the impact of nutritional and inflammatory markers as risk factors for mortality in patients undergoing total or partial hip arthroplasty for hip fractures. A study by Huh et al. investigated 284 older adult patients who underwent hemiarthroplasty for displaced femoral neck fractures (FNF).^20^ Their study has found that patients with a low lymphocyte to CRP ratio and patients with albumin levels <3.75 had an increased 2-year mortality rate. Another systematic review by Pan et al. examined the impact of hematologic inflammatory markers on the prognosis of geriatric hip fractures, concluding that factors such as an elevated neutrophil-to-lymphocyte ratio are associated with increased long-term mortality.^21^ Further studies demonstrate an association between elevated CRP and a low albumin ratio, as well as higher mortality, even after elective THA surgery.^22^^,^^23^ This study corroborates with the current literature, as it observed that lower postoperative albumin levels and higher CRP levels were significantly associated with increased mortality at various postoperative intervals. These results underscore the importance of incorporating these markers into preoperative evaluations to stratify patient risk better and potentially guide perioperative management strategies.

Patient-related factors such as comorbidities, renal function, and age have been shown to significantly influence mortality following both hemiarthroplasty and total hip arthroplasty for femoral neck fractures. Prior research has consistently demonstrated that a higher burden of comorbidities, as measured by the Charlson Comorbidity Index (CCI), is associated with poorer outcomes after hip fracture surgery.^24^ Hailer et al. have investigated early mortality after THA in patients with FNF.^16^ Overall, 24,699 patients were matched with 118,518 controls. The authors have found that a Charlson comorbidity index of 3 or more, an American Society of Anesthesiologists (ASA) grade of 3 and above, male sex, an age of 80 years and above, an income below the first quartile, and lower level of education were associated with an increased risk of 90-day mortality. The authors concluded that patients with no substantial comorbidity and an age of less than 80 years have a lower risk of early death. This study's results align with current literature, as higher CCI scores, impaired renal function, and the presence of CKD were all associated with elevated mortality risk at 90 and 180 days, particularly in the hemiarthroplasty group. Age also emerged as a significant predictor of mid-term mortality, aligning with well-established evidence that advanced age reduces physiological resilience and increases surgical risk. These results highlight the importance of thorough preoperative evaluation to identify high-risk individuals and tailor surgical decision-making accordingly.

## 5 Limitations

This study has several limitations. First, as a retrospective analysis, it is inherently subject to selection bias and may not account for all potential confounding variables, despite the use of multivariate analyses. Second, while the study analyzed a large patient population, it was conducted in a single tertiary trauma center, which may limit the generalizability of the findings to other institutions or healthcare settings. Additionally, some laboratory and clinical parameters were not consistently available for all patients, which may have introduced information bias. Furthermore, the thresholds for albumin, creatinine, and other laboratory values derived from the ROC curve analyses should be interpreted with caution, as they are specific to this cohort and may not be applicable universally.

## 6 Conclusion

Advanced age, elevated inflammatory markers, impaired renal function, and indicators of poor nutritional status were found to be significant risk factors for mortality in patients undergoing arthroplasty following femoral neck fractures. In the THA group, postoperative albumin levels ≤2.85 g/dL were associated with increased 30-day mortality, while CRP levels >19.15 mg/dL predicted higher mortality at both 90 and 180 days. In the hemiarthroplasty group, WBC counts above 14.48 × 10^9/L, serum albumin levels below 3.55 g/dL, and Charlson comorbidity scores above 7.5 were associated with increased 30-day mortality. At 90 and 180 days, mortality risk remained elevated in patients with age over 83.65 and 89.34 years, respectively, WBC >13.49, albumin <3.35–3.45 g/dL, creatinine >1.08 mg/dL, and Charlson score >6.5. These findings suggest that early identification of these markers may improve risk stratification and guide perioperative decision-making.

## Patients consent

Non applicable.

## Author contribution

**Itay Ron, MD** – Conceptualization, Writing – Original Draft, Data Analysis, Interpretation.**Lizi Tamam, BA** – Data Curation, Data Management.**Bezalel Peskin, MD** – Methodology, Study Design.**Nabil Ghrayeb, MD** – Methodology, Study Design.**Doron Norman, MD** – Project Administration, Supervision.**Jacob Shapira, MD** – Writing – Review & Editing, Supervision, Overall Project Oversight.

## IRB approval statement

This study was performed in accordance with the ethical standards in the 1964 Declaration of Helsinki. This study was carried out in accordance with relevant regulations of the US Health Insurance Portability and Accountability Act (HIPAA). Details that might disclose the identity of the subjects under study have been omitted. This study was approved by the IRB.

## IRB approval

This document is an English attest document to the attached IRB approval written in Hebrew.

Rambam medical center's Helsinki committee approved the conduct of medical study under the name ".

The approval was given by the institutional committee at January 11, 2021.

Chairman of the committee Prof. Shimon Pollak gave the approval to the main investigator Dr. Jacob Shapira.

## Funding

Non applicable.

## Declaration of competing interest

• **Itay Ron, MD** – The author declares no conflicts of interest relevant to this work.
• **Lizi Tamam, BA** – The author declares no conflicts of interest relevant to this work.
• **Bezalel Peskin, MD** – The author declares no conflicts of interest relevant to this work.
• **Nabil Ghrayeb, MD** – The author declares no conflicts of interest relevant to this work.
• **Doron Norman, MD** – The author declares no conflicts of interest relevant to this work.
• **Jacob Shapira, MD** – The author declares no conflicts of interest relevant to this work.
